# Potential contribution of strigolactones in regulating scion growth and branching in grafted grapevine in response to nitrogen availability

**DOI:** 10.1093/jxb/ery206

**Published:** 2018-05-30

**Authors:** Noé Cochetel, Eloïse Météier, Isabelle Merlin, Cyril Hévin, Jean-Bernard Pouvreau, Pierre Coutos-Thévenot, Michel Hernould, Philippe Vivin, Sarah Jane Cookson, Nathalie Ollat, Virginie Lauvergeat

**Affiliations:** 1 EGFV, Bordeaux Sciences Agro, INRA, Université de Bordeaux, Villenave d’Ornon, France; 2 LBPV, Laboratoire de Biologie et de Pathologie Végétales, EA 1157, SFR 4207 QUASAV, UFR Sciences et Techniques, Université de Nantes, Nantes, France; 3 SEVE, Laboratoire Sucres & Echanges Végétaux-Environnement, UMR Ecologie et Biologie des Interactions CNRS 7267, Université de Poitiers, Poitiers, France; 4 BFP, INRA, Université de Bordeaux, Villenave d’Ornon, France

**Keywords:** Grafting, grapevine, nitrogen, rootstocks, shoot branching, strigolactones

## Abstract

In grafted plants, rootstocks assure the mineral nutrition of the scion and modify its development. In this study, we show that two grapevine rootstock genotypes have different shoot branching architectures when cultivated as cuttings and that this trait is transmitted to the scion when grafted. Shoot branching plasticity in response to nitrogen supply was also studied. As strigolactones are known to have a role in the regulation of shoot development in response to nutrient availability, their involvement in the control of scion architecture by the rootstock was investigated. Functional characterization of putative grapevine strigolactone biosynthetic genes in Arabidopsis mutants or grapevine cell suspensions showed similar functions to those of Arabidopsis. Both rootstocks produced strigolactone-like compounds; the quantity produced in response to nitrogen treatments differed between the two rootstock genotypes and correlated with the expression of putative strigolactone biosynthetic genes. Exudation of strigolactone-like compounds by both rootstocks was closely related to the developmental pattern of the scion in grafted plants. These results suggest that differential regulation of strigolactone biosynthesis in response to nitrogen availability may contribute to the control of scion development conferred by each rootstock genotype.

## Teaser

Grapevine rootstocks regulate scion growth and architecture differently in response to nitrogen availability. Strigolactones are suggested as key root-derived molecules modulating scion development in grafted grapevine.

## Introduction

In the context of climate change and the development of sustainable agriculture, understanding how crop growth is controlled is a primary objective for plant research. Among the mechanisms that have evolved to enable plants to adapt to environmental changes, the plasticity of plant architecture allows them to adjust their development to optimize growth. The control of root and shoot branching is an example of such an adaptive strategy. While the basic plan of a plant is established during embryogenesis, its architecture, and particularly the branching pattern, can be adjusted in response to environmental variations. Nutrient deficiency, for example, is known to reduce the shoot/root mass ratio through an increase in root growth together with a decrease in shoot branching (Domagalska and Leyser, 2011; Poorter *et al.*, 2012). Hormones are well-known regulators of shoot branching. They are involved in the long-distance signalling established between the shoots and roots to integrate internal plant nutrient status and the perception of heterogeneous external resources (Rameau *et al.*, 2015; Teichmann and Muhr, 2015). While the roles of auxin and cytokinins have long been described in the literature, more recently a number of functions have also been ascribed to strigolactones (SLs), including involvement in the complex hormonal regulation network of shoot branching (Gomez-Roldan *et al.*, 2008; Umehara *et al.*, 2008).

SLs were first shown to promote seed germination of parasitic plants such as *Striga* and *Orobanche* spp. (Cook *et al.*, 1966), and to promote arbuscular mycorrhizal symbiosis (Akiyama *et al.*, 2005). SLs are now known to be involved in several processes controlling plant development in response to nutrient availability, and particularly for their role in the inhibition of shoot branching (Xie *et al.*, 2010; Marzec *et al.*, 2013; Waldie *et al.*, 2014; Lopez-Obando *et al.*, 2015; Pandey *et al.*, 2016). Studies using grafting experiments identified many actors involved in the SL signalling pathway and demonstrated that SLs are mainly synthesized in the roots and move acropetally to control shoot development (Dun *et al.*, 2009; Xie *et al.*, 2010). Carlactone, the direct precursor of SLs, is derived from the carotenoid pathway and is produced in three steps catalysed by an isomerase, D27 (Dwarf 27 in rice), and two dioxygenases, CCD7 and CCD8 (Carotenoid Cleavage Dioxygenase 7/8) (Lopez-Obando *et al.*, 2015). MAX1 (More Axillary Growth 1), a member of the cytochrome P450 family, and its orthologues and paralogues are known to be involved in the last steps of SL biosynthesis from carlactone (Booker *et al.*, 2005; Seto *et al.*, 2014). Downstream of perception, different actors in the signalling pathway have been identified, such as BRC1 (BRANCHED 1), which was described as a putative integrator in the control of bud outgrowth (Rameau *et al.*, 2015). 

The induction of SL synthesis during nutrient starvation plays an important role in the developmental plasticity of plants (Kapulnik and Koltai, 2014; Pandey *et al.*, 2016). It has been shown that the increase in SL production during phosphorus starvation is correlated with inhibition of shoot branching and an increase in lateral root growth (Brewer *et al.*, 2013). Nitrogen (N) limitation also increases SL synthesis depending on the species (Yoneyama *et al.*, 2012; Brewer *et al.*, 2013). More recently, de Jong *et al.* (2014) described the mechanisms of hormonal signalling involved in inhibition of shoot branching in Arabidopsis during N starvation. They demonstrated the influence of SLs on biomass allocation and the importance of the systemic signalling network established with auxin and cytokinins.

In grapevine, like other species that are cultivated by grafting (Warschefsky *et al.*, 2016), responses to environmental cues require communication between the two different genotypes, that is, the scion and the rootstock. Cultivation of the European grapevine species *Vitis vinifera* requires grafting on to phylloxera-resistant rootstocks of American origin throughout most of the world (May, 1994). Rootstock genotypes are also selected for their ability to adapt to abiotic soil conditions and other pathogens. In addition, rootstocks may confer several agronomical traits to the scion, for example, to modify its development, precocity, productivity, and fruit quality. Since the establishment of an equilibrium between vegetative growth and berry quality is a core concept in viticulture, many studies have been performed to elucidate the communication that is established between the scion and the rootstock (Aloni *et al.*, 2010; Cookson *et al.*, 2014; Yang *et al.*, 2015). Scion vegetative growth may be affected depending on the rootstock genotype, a process often called ‘conferred vigour’ (Ollat *et al.*, 2003; Zhang *et al.*, 2016); this can be defined by total pruning wood weight in the vineyard or by shoot dry weight (DW) in pot experiments trained to one main stem (Lecourt *et al.*, 2015; Rives, 2000). However, these measurements do not provide information on the scion architecture and its development. Furthermore, the mechanisms involved in the control of scion growth by the rootstock in grapevine are still unknown.

Hormones are graft-transmissible actors that potentially control scion development depending on the rootstock genotype (Albacete *et al.*, 2015). Rootstocks could modulate the rate of berry ripening through the auxin pathway (Corso *et al.*, 2016) and modify abscisic acid-mediated responses to water deficit (Rossdeutsch *et al.*, 2016). The production of hormones by grapevine rootstock genotypes has been studied for a long time; Skene and Antcliff (1972) highlighted the primordial role of these root-derived signals in scion-growth control. Nikolaou *et al.* (2000) showed that xylem cytokinins in the shoot are dependent on the rootstock genotype and are correlated with bud burst and shoot elongation. Van Hooijdonk *et al.* (2011) proposed that dwarfing rootstocks in apple could reduce the basipetal transport of auxin to the roots, which in turn decreases the transport of cytokinin and gibberellin from the roots to the scion. These data demonstrate the importance of hormone signalling in the control of scion growth by the rootstock. Several authors have suggested that, as well as the well-known crosstalk between auxin and cytokinins, other actors could contribute to the regulation of scion architecture (Aloni *et al.*, 2010; Van Hooijdonk *et al.*, 2009; Van Hooijdonk *et al.*, 2011).

Previous studies using two grapevine rootstocks, *Vitis riparia* cv. Riparia Gloire de Montpellier (RGM) and the *Vitis berlandieri* × *Vitis rupestris* hybrid cv. 1103 Paulsen (1103P), which are known to confer low and high scion vigour, respectively, showed that RGM was more responsive to N availability than 1103P in terms of regulation of scion growth and gene expression in the roots (Lecourt *et al.*, 2015; Cochetel *et al.*, 2017). As the rootstock genotype constitutes the root system of a grafted plant, differences in SL biosynthesis and/or transport could differentially regulate scion development in response to N supply. The main objective of this work was to study the regulation of the SL pathway in two grapevine rootstocks and determine the contribution of these hormones in the rootstock control of scion growth. The study aimed (i) to characterize the growth and shoot architecture of 1103P and RGM when grown as cuttings and their responses to different levels of N supply; (ii) to characterize the growth and shoot architecture of a *V. vinifera* scion when grafted on these two rootstocks; and (iii) to determine whether rootstock effects on scion growth and shoot architecture are correlated with differences in regulation of the SL pathway.

## Materials and methods

### Plant material and growth conditions

Grapevines were grown as either cuttings (RGM and 1103P) or grafted plants with *V. vinifera* cv. Cabernet Sauvignon (CS) as the scion; the two scion/rootstock combinations are named CS/RGM and CS/1103P.

In a previous study, a split-root experiment was conducted to investigate the root transcriptomic responses of CS/RGM and CS/1103P to a heterogeneous N supply (Cochetel *et al.*, 2017). Briefly, a double-grafting system was used to obtain two-roots-one-shoot plants (Tandonnet *et al.*, 2010). Grafted plants resembled an inverted ‘Y’ and were cultivated in two sand-filled pots (one per root system) in a greenhouse. A low-nitrate (LN) nutrient solution [0.8 mM KNO_3_, 0.57 mM K_2_HPO_4_, 0.69 mM MgSO_4_, 1.39 mM CaCl_2_, 0.8 mM K_2_SO_4_, 0.3 mM CaSO_4_, and micronutrients as described by Lecourt *et al.* (2015)] was supplied for 2 weeks. At the beginning of the experiment, one side of the root system was irrigated with 5 mM nitrate (HN) solution and the other side was irrigated with LN solution. The root tips from each side (HN and LN) of three plants of CS/RGM and CS/1103P were sampled at 0, 3, and 24 h post treatment (hpt).

For experiments with cuttings and single grafts in a greenhouse, CS was omega grafted (Tandonnet *et al.*, 2010) on to 1103P or RGM to obtain CS/1103P and CS/RGM. After callusing, these grafted plants, as well as cuttings of 1103P and RGM, were rooted and transferred to sand-filled pots in the greenhouse and irrigated for 50 d with a full nutrient solution (N=4.82 mM) (Cookson *et al.*, 2012). At the end of this acclimation period, cuttings were divided into two equal groups. The first group of plants was continuously irrigated for 45 d with LN nutrient solution (0.8 mM N) and the second was supplied with HN solution (5 mM N). After the acclimation period, CS/1103P and CS/RGM were cultivated under HN conditions for 45 d. Leaves, scion stems, roots, and root tips were sampled from CS/1103P and CS/RGM plants cultivated for 1 month in sand-filled pots in the greenhouse and irrigated during the last 2 weeks with LN solution, for further transcript abundance quantification analysis.

**Fig. 1.**
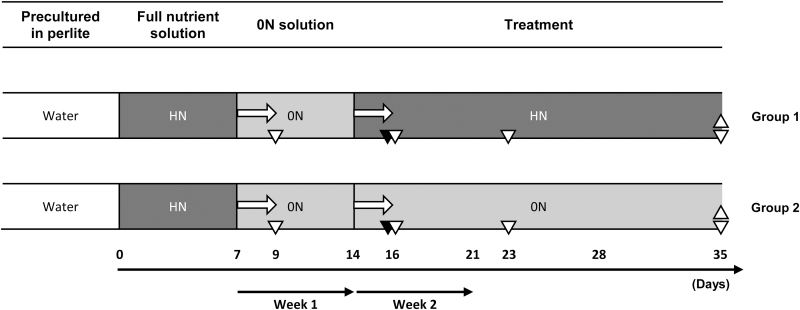
Design of the hydroponic culture experiments. Acclimated plants were grown hydroponically with a full nutrient (HN) solution for 1 week and then transferred into N-free (0N) solution. One week later, plants were divided in two groups: group 1 plants were re-transferred to HN solution and group 2 plants were maintained in 0N solution. For gene expression profiling, root samples were harvested at 0, 3, 12, 24, and 48 hpt (horizontal arrows) from day 7 and day 14 for both groups. The phenotypic characterization was performed on the last day of the experiment (indicated by upward-pointing triangles). The experimental design was the same for cuttings and for grafted plants, except that root exudates were collected only on day 16 of the experiment for the cuttings (black downward-pointing triangles) and on days 9, 16, 23, and 35 for the grafted plants (white downward-pointing triangles).

For hydroponic cultures, plants were propagated *in vitro* on McCown Woody Plant Medium (Duchefa) supplemented with 1.5% sucrose, 0.27 µM 1-naphthalene acetic acid, and 0.7% agar, in a growth chamber at 25 °C/20 °C and subjected to a photoperiod of 16 h light/8 h dark with a light intensity of 145 µmol photons m^–2^ s^–1^ . To produce micrografted plants, cleft grafting was performed. One-month-old plantlets were acclimated on perlite substrate and irrigated with tap water for 1 month. Plants were transferred into hydroponic jars containing 8 liters of a McCown-derived nutrient solution deprived of vitamins and with 5 mM of NH_4_NO_3_ and 2.35 mM of Ca(NO_3_)_2_, 4H_2_O (HN) for 1 week, followed by 1 week of culture in a solution without any source of N (0N) (corresponding to week 1; Fig. 1). For the latter solution, Ca^2+^ was adjusted by adding CaCl_2_. Plants were then moved to the HN condition (group 1) or kept in the 0N condition (group 2) (corresponding to week 2; Fig. 1). The nutrient solution was renewed three times per week. The entire root systems were collected from three plants at 0, 3, 12, 24, and 48 hpt during week 1 and week 2 for transcript abundance quantification analysis. For the measurements of growth and branching, plants were harvested after 35 d of culture.

### Cloning of the full-length coding sequences of *Vitis D27*, *CCD7*, and *CCD8*

Putative *D27*, *CCD7*, and *CCD8* homologues were identified using a BlastX search, using the corresponding Arabidopsis full protein sequences as queries against the V1 gene models of the grapevine genome Pinot Noir PN40024 (Jaillon *et al.*, 2007) on CRIBI (http://genomes.cribi.unipd.it/grape/). Gene-specific primers were designed on the basis of these *V. vinifera* sequences to amplify the full-length coding sequences of *D27* (*VIT_00s0179g00330*), *CCD7* (*VIT_15s0021g02190*), and *CCD8* (*VIT_04s0008g03380*) from cDNAs derived from RNAs isolated from the roots of both 1103P and RGM (primers are listed in Supplementary Table S1 at *JXB* online). PCR fragments obtained with the Phusion high-fidelity DNA polymerase were cloned using the pGEM®-T easy vector system (Promega) before sequencing (GATC Biotech). The sequences have been deposited in GenBank under accession numbers KX865167 and KX865168 for RGM *CCD7* and *CCD8*, and KX865169 and KX904934 for 1103P *CCD7* and *CCD8*, respectively. For *D27*, the RGM cDNA sequence chosen for vector construction showed 100% identity with the sequence from Pinot Noir.

### Vector construction and Arabidopsis transformation

For overexpression experiments in the Arabidopsis *max3-11* mutant, RGM *CCD7* cDNA was cloned into the pH7WG2D.1 plasmid (Karimi *et al.*, 2002) using the Gateway cloning system (Invitrogen) and inserted into One Shot® *ccd*B Survival™ 2 T1^R^ competent cells. To transform the Arabidopsis *max4-1* mutant, a construct containing the RGM *CCD8* coding sequence under the control of the cauliflower mosaic virus (CaMV) 35S promoter was inserted using GoldenBraid 2.0 into a pDGB2Ω1 binary vector (Sarrion-Perdigones *et al.*, 2013). Before plant transformation, each plasmid was sequenced and inserted into *Agrobacterium tumefaciens* strain GV3101. Arabidopsis Col-0*, max3-11*, and *max4-1* were transformed by the floral dip method (Clough and Bent, 1998). Transgenic plants were selected by sowing seeds on Murashige and Skoog (MS) medium (Murashige and Skoog, 1962) containing hygromycin and kanamycin to select for *CCD7* and *CCD8* overexpression, respectively. For each independent transgenic line, the presence of the transgene was verified by PCR on gDNA using the primers listed in Supplementary Table S1.

After sterilization, seeds of Arabidopsis *max3-11* and *max4-1* mutant lines, Col-0, and transgenic lines of each genotype transformed with the construct allowing the overexpression of either grapevine *CCD7* or *CCD8* were sown on 1/2 MS medium complemented with the appropriate antibiotic. After 10 d, plantlets were transferred to a growth room with a temperature of 22 °C day/18 °C night and a photoperiod of 16 h light/8 h dark.

### Grapevine cell transformation and culture

The three RGM coding sequences of *D27*, *CCD7*, and *CCD8* were each subcloned under the constitutive CaMV 35S promoter in a cassette containing the *NPTII* gene using GoldenBraid 2.0 methodology. The resulting plasmid, pDGB2α1, was introduced into *A. tumefaciens* strain EHA105, which was used to transform an embryogenic cell suspension culture derived from the 41B hybrid rootstock (*V. vinifera* cv. Chasselas × *V. berlandieri*). Transgenic cells were transformed, selected on paromomycin, and maintained according to Coutos-Thévenot *et al.* (2001). Control cells were generated by transformation with empty pDGB2α1 containing only the *NPTII* gene.

### Phenotypic analysis of plant growth and architectural parameters

At the end of the acclimation period (corresponding to day 0) and 45 d after continuous irrigation with either LN or HN solution, between 10 and 15 greenhouse-grown plants were analysed. Leaf N status was evaluated using the Yara N-Tester®. This instrument detects the leaf chlorophyll content, which is related to the N content, by optical measurement. The length of each internode of the main stem, the number of leaves and the total leaf surface, the length and rank of each primary lateral branch (LB I) and the number and length of secondary LBs (LB II; LBs grown under LB I) were recorded. The DW of roots, hardwood cutting, leaves, and stems (including petioles) was evaluated after drying at 70°C for 72 h. Relative growth rates were calculated as described by Hoffmann and Poorter (2002).

At the end of the hydroponic experiments, the number of LB I was counted and the fresh weight (FW) of roots, LBs, and total shoots was measured for each plant.

For transformed Arabidopsis plants, after 1 month of culture in a growth chamber, the number of primary rosette branches and primary cauline branches of 10 individual plants per genotype was counted.

### Transcript level quantification

Total RNA was isolated from root samples as described by Cookson *et al.* (2013). Total RNA (1.5 µg) was reverse transcribed into cDNA using the SuperScript III First-Strand Synthesis System for reverse transcription (RT)–PCR (Invitrogen). Quantitative (q) PCRs were performed using SYBR Green on an iCycler iQH (Bio-Rad) according to the procedure described by the supplier, with 0.2 µM of primers for each studied gene. Gene expression was calculated either as normalized expression (2^–∆ct^) or as relative normalized expression with the 2^–∆∆ct^ method (Livak and Schmittgen, 2001), with the reference genes *EF1γ* and *GAPDH* for normalization in grapevine, and *ACT2* and *UBC21* in Arabidopsis. Primer sequences are listed in Supplementary Table S1. *MAX1*, *D14*, *PDR1*, *BRC1*, and *MAX2* were identified as genes encoding putative protein sequences showing the highest similarity with known proteins from different species.

### Exudate sampling and *P. ramosa* seed germination assays

Root exudates from hydroponically grown plants were sampled at different time points (Fig. 1). Five plants from each condition were transferred to 50 ml tubes containing the same nutrient solution to which they had been exposed during the treatment, covered with aluminium foil, and incubated for 5 hours in the growth chamber. For the grapevine cell suspensions, 1 ml of the liquid medium was collected 2 days after having been renewed. The exudates were filtered through 0.2 µm filters and stored at 4 °C in the dark. The germination-stimulant activity of exudates on seeds of *Phelipanche ramosa* L. Pomel was measured using 3-(4,5-dimethylthiazol-2-yl)-2,5-diphenyltetrazolium bromide assays as described by Pouvreau *et al.* (2013) at the Laboratoire de Biologie et Pathologie Végétales (Nantes, France). Racemic GR24 was used at a concentration of 1.10^–7^ M as a positive control and achieved 62–70% of seed germination across all the experiments. The absorbance results were normalized by subtracting the negative control value (parasitic seeds incubated without germination stimulant, <1% germination in all the experiments) from each measured value. Data were converted into percentage germination and are reported as a percentage relative to the positive control GR24.

### Statistical analyses

All statistical analyses were performed using the software R (R Core Team, 2016). For comparisons between two means, Student’s *t*-test was used with Bonferroni correction. When assumptions for parametric tests were not respected, the Wilcoxon non-parametric test was used. For comparisons between more than two means, a Tukey’s multiple test was used after one-way ANOVA. When assumptions for parametric tests were not respected, a multiple comparison test was performed after a Kruskal-Wallis test using the function kruskalmc from the pgirmess R package. Letters to indicate significant differences among multiple comparisons were obtained using the function multcompLetters from the R package multcompView. For the variable main stem number, a Fisher’s exact test was performed based on a contingency table indicating whether there was a unique main stem or not. For the variable LB number/node number, the difference in the percentage of outgrown axillary bud between two samples was determined using the chi-square test.

## Results

### Up-regulation of SL-related gene expression in the roots of CS/RGM in response to N availability

**Fig. 2.**
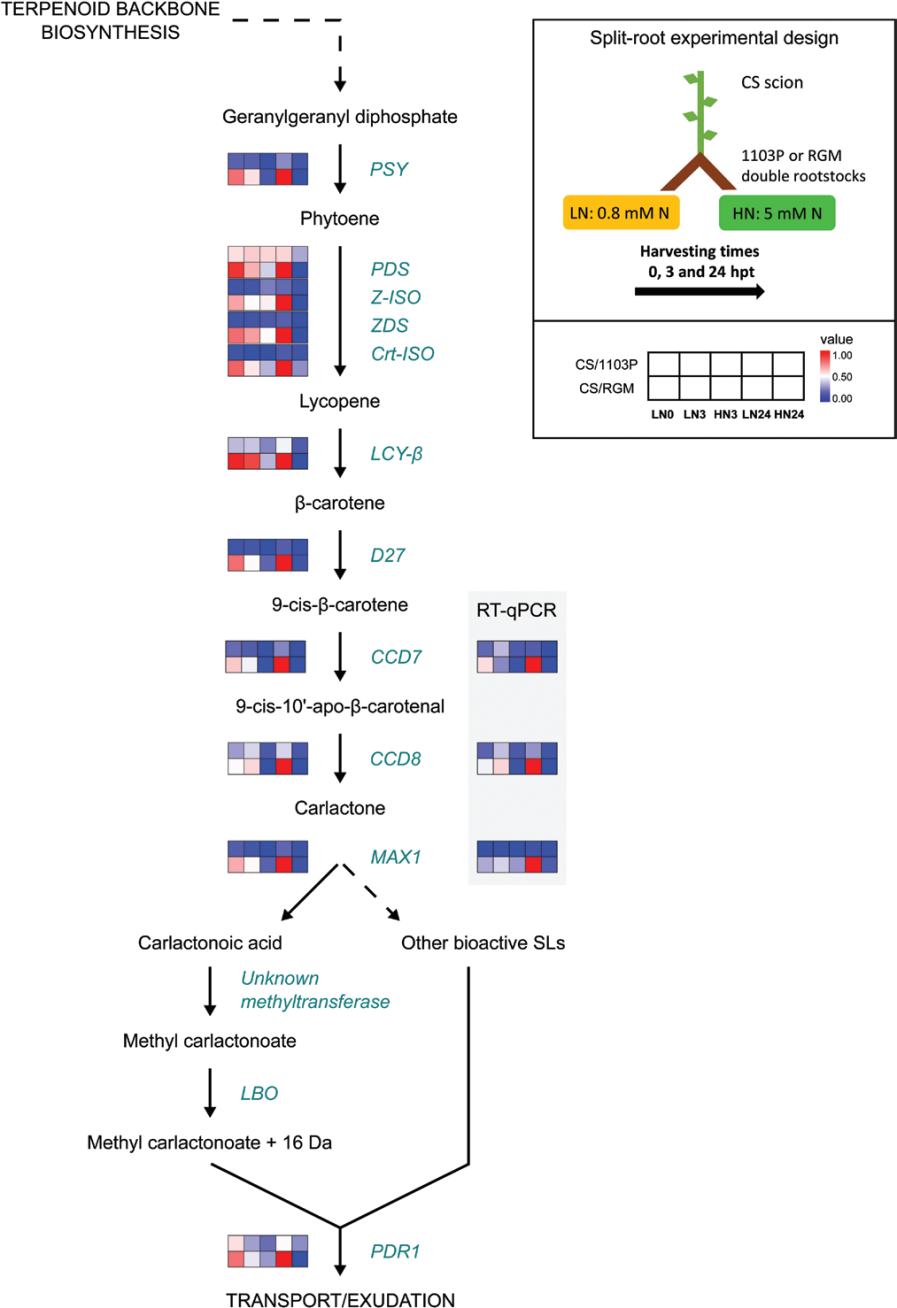
Genes encoding enzymes of the SL biosynthetic pathway are differentially regulated in roots of CS/RGM in response to N availability. Transcript levels are presented as heatmaps of mean values of reads per kilobase per million mapped reads (RPKM; RNA-seq) or normalized values (RT–qPCR) for each condition as described in the map (top right). For each gene, data were transformed to range from 0 (blue, minimum value) to 1 (red, maximum value). RPKM values were obtained from RNA-seq data in Cochetel *et al.* (2017). Mean values of normalized expression obtained using RT–qPCR are presented for putative *CCD7*, *CCD8*, and *MAX1* genes, *n*=3 individual plants. LN0, roots at 0 hpt (grown with LN nutrient solution); LN3 and LN24, roots from the side with LN solution harvested at 3 and 24 hpt, respectively (after the addition of HN solution to the other side); HN3 and HN24, roots harvested at 3 and 24 hpt on the side to which HN was added. *CCD7*, *CAROTENOID CLEAVAGE DIOXYGENASE 7* (*9-CIS-BETA-CAROTENE 9ʹ,10ʹ-CLEAVING DIOXYGENASE*); *CCD8*, *CAROTENOID CLEAVAGE DIOXYGENASE 8* (*ALL-TRANS-10ʹ-APO-BETA-CAROTENAL 13,14-CLEAVING DIOXYGENASE*); *Crt-ISO*, *PROLYCOPENE ISOMERASE*; *PSY*, *PHYTOENE SYNTHASE*; *D27*, *DWARF27* (*BETA-CAROTENE ISOMERASE*); *LBO*, *LATERAL BRANCHING OXIDOREDUCTASE*; *LCY-β*, *LYCOPENE BETA-CYCLASE*; *MAX1*, *MORE AXILLARY BRANCHES 1* (*CYP711A1*); *PDR1*, *PLEIOTROPIC DRUG RESISTANCE 1*; *PDS*, *PHYTOENE DESATURASE*; *ZDS*, *ZETA-CAROTENE DESATURASE*; *Z-ISO*, *ZETA-CAROTENE ISOMERASE*.

Our recent root transcriptomic study on the response of CS/1103P and CS/RGM to heterogeneous N availability identified an SL-related module using weighted gene co-expression network analysis (Cochetel *et al.*, 2017). The RNA-seq data on 12 genes putatively involved in the biosynthetic pathway of the SL precursor β-carotene and the SL compounds contained in that module are shown in Fig. 2. RT–qPCR validation of the expression profiles of putative *CCD7*, *CCD8*, and *MAX1* genes was performed on roots from the same split-root experiment (Fig. 2). Quantification of transcript abundance showed that all these genes were significantly differentially expressed only in CS/RGM, with higher transcript accumulation at 24 hpt in the LN-supplied roots compared with the HN-supplied roots (Fig. 2). Altogether, these data showed that genes characterized as SL related were differentially regulated in roots subjected to a heterogeneous N supply in a rootstock genotype-dependent manner.

### Functional characterization of grapevine SL-related genes

Putative CCD7 and CCD8 encoding sequences were cloned from cDNA obtained from root RNAs of the RGM and 1103P genotypes. Each sequence showed more than 97% identity to the corresponding *V. vinifera* sequences at the nucleotide level and more than 98% of identity at the amino acid level. Amino acid sequences deduced from cDNA sequences of *Vitis* exhibited around 60% identity and 75% similarity with Arabidopsis AtMAX3 and AtMAX4 proteins (data not shown).

**Fig. 3.**
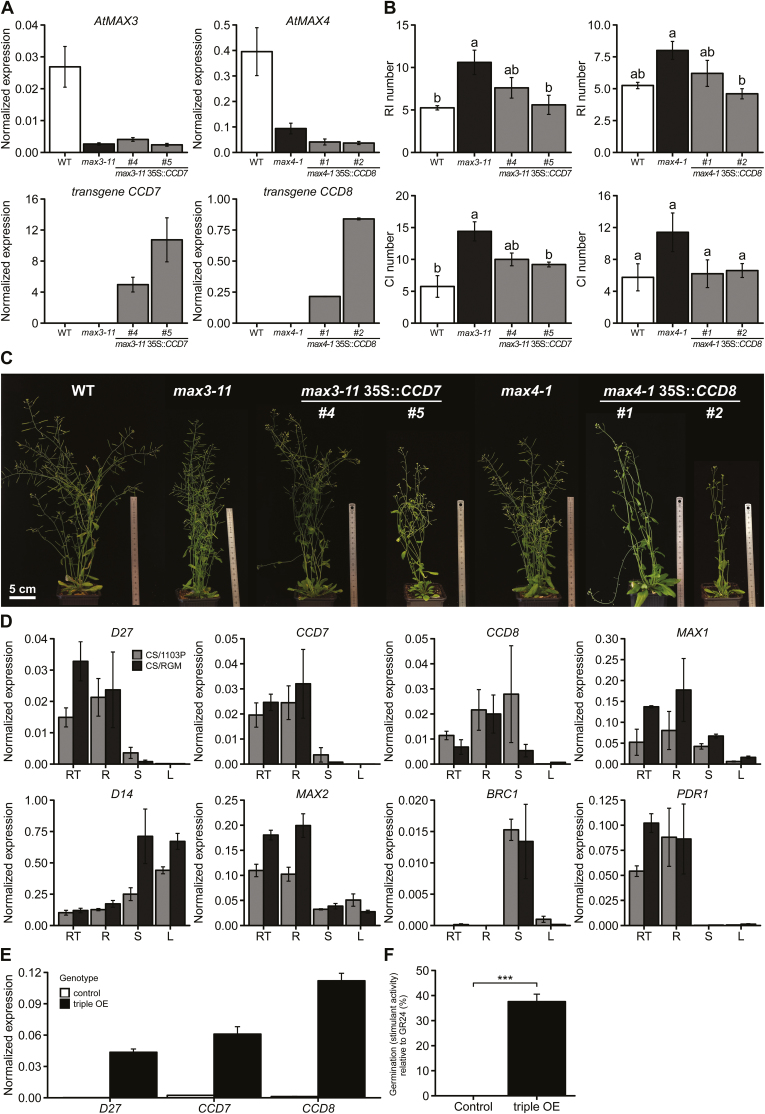
Functional characterization of grapevine SL-related genes. (A–C) Transformation of Arabidopsis *max3-11* and *max4-1* mutants with grapevine *35S::CCD7* or *35S::CCD8* genes, respectively. (A) Quantification of endogenous (*AtMAX3* or *AtMAX4*) and transgene (*CCD7* or *CCD8*) transcript levels in wild-type (Col-0), mutants, and two independent transgenic lines in the corresponding mutant background. RT–qPCR results from RNA isolated from shoots of 40-day-old seedlings are presented as normalized expression. Data are presented as means ±SE, *n*=3 individual plants. (B) Number of primary rosette branches (RI) and primary cauline branches (CI) of Col-0, *max3-11*, *max4-1*, and transgenic lines. Data are presented as means ±SE, *n*=10 individual plants. Letters above the bars indicate significant differences between genotypes as determined by Tukey’s multiple test (*P*<0.05). (C) Shoot branching phenotypes of Col-0, mutants, and transgenic lines. (D) Expression profiles of genes involved in SL biosynthesis (*D27*, *CCD7*, *CCD8*, *MAX1*), transport (*PDR1*), and signalling (*D14*, *MAX2*, *BRC1*) in different organs of 1-month-old CS/1103P and CS/RGM plants cultivated in a greenhouse with a low N content solution (0.8 mM NO_3_ ^–^). L, leaves; R, roots; RT, root tips; S, stem. RT–qPCR results are presented as normalized expression. Data are presented as means ±SE, *n*=3 individual plants. (E, F) Overexpression of grapevine *D27*, *CCD7*, and *CCD8* genes in 41B suspension cells. (E), Normalized expression of endogenous and transgenes in control and *35S::D27*//*35S::CCD7*//*35S::CCD8* (triple OE) transgenic cells. Data are presented as means ±SE, *n*=3 individual plants. (F) Activity of extracellular medium from control and transgenic (triple OE) cell suspensions (diluted 1:2) on *P. ramosa* seed germination. Germination-stimulant activities are represented as a percentage relative to the positive control GR24. Data are presented as means ±SE, *n*=9 (three biological replicates repeated three times). ***P<0.001, Student’s *t*-test.

Arabidopsis *Atmax3-11* and *Atmax4-1* mutants were transformed with the corresponding RGM sequences under the control of the CaMV 35S promoter. The transcript abundance of endogenous *AtMAX3* or *AtMAX4* and the corresponding transgenes were confirmed by RT–qPCR (Fig. 3A). Phenotypic analysis of two independent lines for each transformation relative to Col-0 and mutant lines showed that overexpression of RGM *VrCCD7* or *VrCCD8* in the corresponding mutant genetic background partly reverted the *max3* or *max4* phenotype (Fig. 3C). While both mutant genotypes presented a significantly higher number of primary rosette and primary cauline branches compared with the wild type (WT) (Fig. 3B), the transgenic lines followed a branching behaviour more similar to that of the WT. These phenotypes correlated with the expression level of the transgenes.

The expression pattern of SL biosynthesis and signalling pathway genes was investigated in grafted grapevines cultivated in the greenhouse (Fig. 3D). As expected, transcripts of putative *D27*, *CCD7*, *CCD8*, *MAX1*, and *PDR1* genes were more abundant in the roots than in stems and leaves. Transcripts of the *MAX2* putative orthologue were also highly accumulated in the roots, while putative *D14* and *BRC1* transcripts were more highly accumulated in the stems and leaves.

Overexpression experiments were performed in grapevine suspension cells transformed with a unique construct containing *35S::VrD27*, *35S::VrCCD7*, and *35S::VrCCD8*. After stabilization of the cell cultures, bioassays were performed to elucidate the potential ability of the compounds secreted into the external medium by the cells to trigger the germination of SL-inducible *P. ramosa* L. Pomel seeds. The extracellular medium of the cells overexpressing the three genes (Fig. 3E) was clearly able to induce *P. ramosa* seed germination (indicating the presence of SL molecules in the medium) in comparison to the control medium containing cells transformed with the empty vector (Fig. 3F). These results demonstrated that the SL-related gene orthologues in grapevine encode enzymes that are involved in the SL biosynthetic pathway and that they could trigger the production of SL.

### N starvation decreases shoot growth and induces expression of SL-related genes and exudation of compounds triggering *P. ramosa* seed germination

**Fig. 4.**
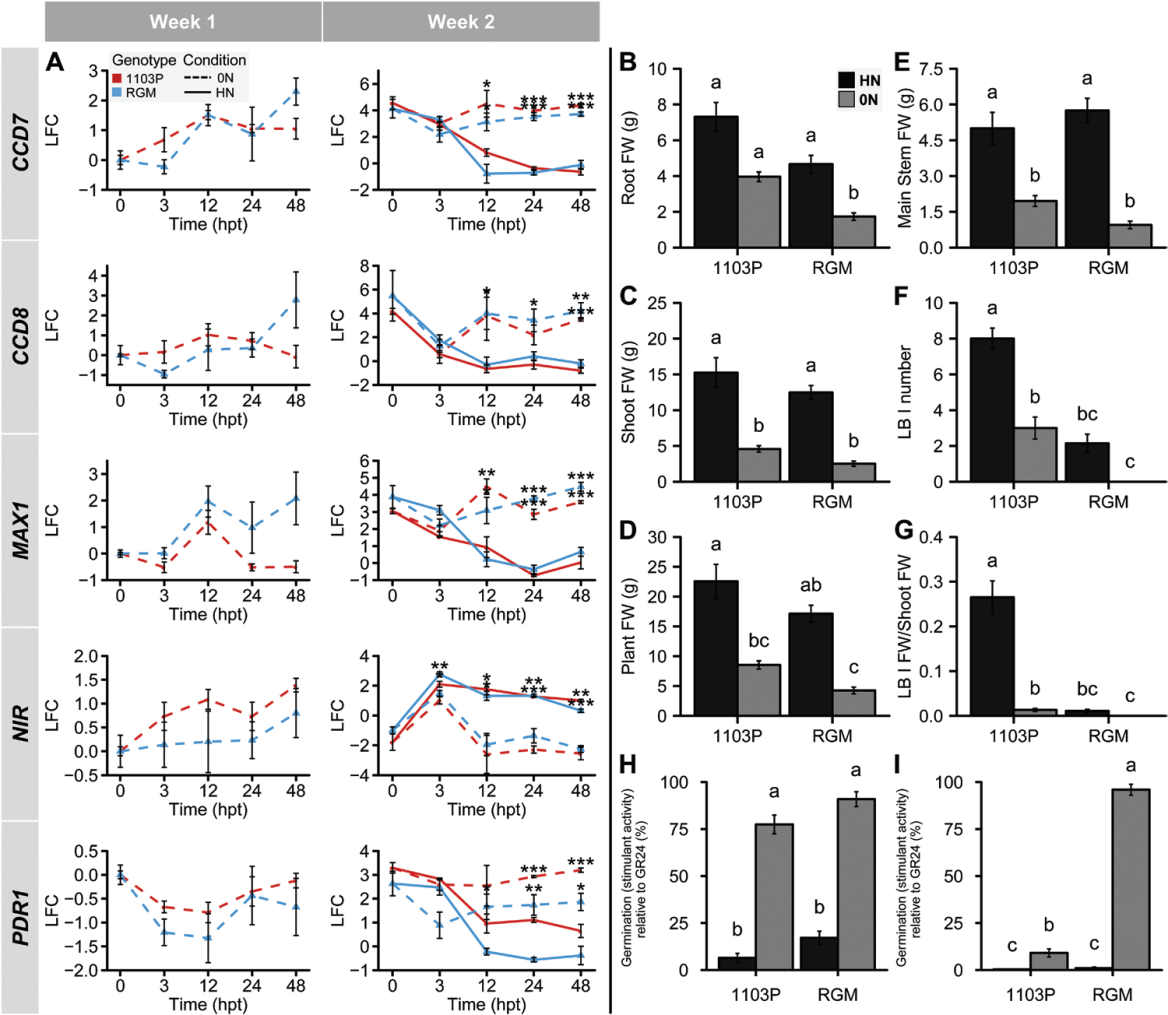
Responses to N availability of RGM and 1103P grown as cuttings in hydroponic culture. (A) Transcript levels of SL-related and *NIR* genes in roots of 1103P (red) and RGM (blue) cuttings during weeks 1 and 2 of N treatment. Dashed lines represent samples from 0N plants and solid lines correspond to HN resupplied plants. Relative normalized expression values are represented as log_2_ fold change (LFC) relative to the beginning of the treatment (0 hpt, week 1). Data are presented as means ±SE, *n*=3 individual plants. Asterisks represent significant differences between 0N and HN conditions for each genotype using Student’s *t*-test: *P<0.05, **P<0.01, ***P<0.001. (B–G) Fresh weights of different plant organs measured after 35 d of N treatments. Lateral branches (LB) fresh weight per shoot fresh weight (G) were calculated. Data are presented as means ±SE, *n*=15 individual plants. Letters above the bars indicate significant differences between treatments (i.e. condition × genotype) using a multiple comparison test after Kruskal-Wallis (*P*<0.05). (H) Germination-stimulant activities of root exudates diluted 1:2. Exudates were collected during the second week of culture from group 1 and group 2 plants. Activities are presented as a percentage relative to the positive control GR24. Data are presented as means ±SE, *n*=20 (exudates from five individual plants repeated four times). (I) The same root exudates samples were diluted 1:10 and re-plated. Letters indicate significant differences between treatments (i.e. condition × genotype) using a Tukey’s test (*P*<0.05).

Under hydroponic culture conditions, the transfer of 1103P and RGM cuttings to HN solution (Fig. 1, group 1, week 2) triggered the sustained accumulation of the transcript encoding nitrite reductase (*NIR*); the level of *NIR* transcripts was lower and decreased over time in plants cultivated with 0N solution (Fig. 4A). Transfer of plants to N-free solution induced the expression of *CCD7*, *CCD8*, and putative *MAX1*. Interestingly, a continuous increase in SL biosynthesis gene expression was observed between weeks 1 and 2 for both genotypes. The induction of expression of these three genes was slightly higher in RGM than in 1103P roots at 48 hpt in week 1. These results indicate induction of the SL-related genes in both genotypes in response to N starvation.

When both genotypes from group 1 (subjected to HN) were compared, no significant difference was observed for root or shoot FW, resulting in plants with the same total FW at day 35 (Fig. 4B–D). However, the developmental pattern was different, with a significantly higher number of LB I and a higher corresponding FW (relative to whole shoot FW) for 1103P (Fig. 4F, G). For both rootstock genotypes, plant growth in 0N conditions (Fig. 1, group 2) was reduced (Fig. 4B–E). This effect was more pronounced in RGM, with whole plant FW reduced approximately 4-fold, while the growth reduction of 1103P was 2.6-fold (Fig. 4D). LB I production was abolished for RGM in the 0N condition, while a decrease was observed for 1103P (Fig. 4F, G). N starvation conditions triggered a reduction of LB growth in both genotypes.

Root exudates collected from N-starved RGM and 1103P plants (diluted 2-fold) were more able to induce germination of *P. ramosa* seeds (Fig. 4H) than exudates produced by HN-supplied plants. This induction correlated with a higher expression of putative *PDR1* in the roots of N-starved plants (Fig. 4A). Interestingly, exudates diluted 10-fold from RGM induced a higher percentage of *P. ramosa* seed germination than those from 1103P (Fig. 4I).

Altogether, these results showed that N deficiency had a strong effect on the shoot architecture of both RGM and 1103P. Exudation of SL-like compounds was induced by N starvation in both rootstocks. These SL-like compounds produced by the roots may also be transported to the shoot and could explain the inhibition of LB I growth of 1103P and RGM cuttings.

### Scion branching is differently regulated by the rootstock in response to N availability in grafted plants

**Fig. 5.**
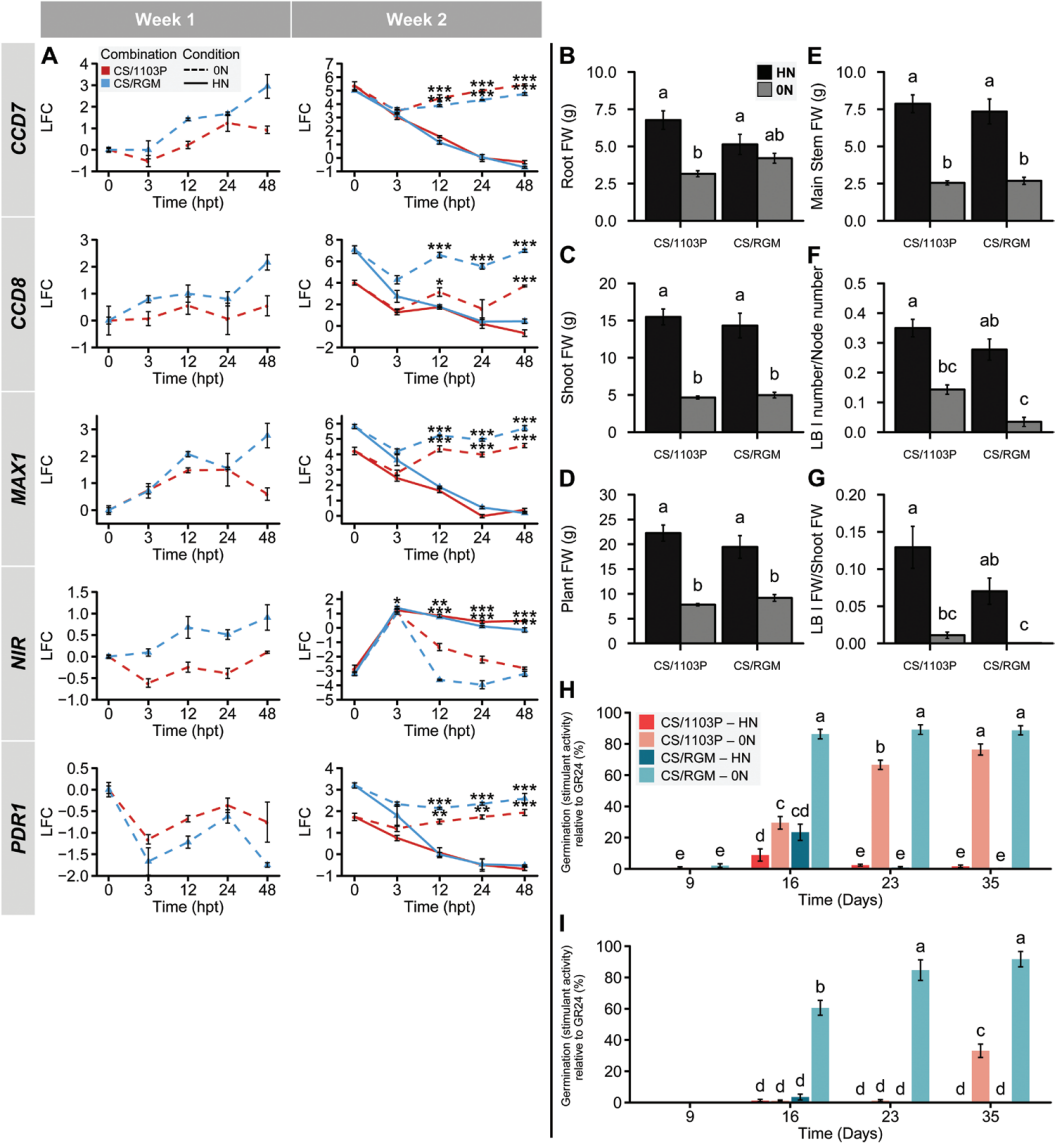
Responses of the CS/RGM and CS/1103P combinations to N availability in hydroponic cultures. (A) Expression of SL-related and *NIR* genes in roots of 1103P (red) and RGM (blue) cuttings during weeks 1 and 2 of treatment. Dashed lines represent samples from 0N plants and solid lines correspond to HN resupplied plants. Relative normalized expression values are represented as log_2_ fold change (LFC) relative to the beginning of the treatment (0 hpt). Data are presented as means ±SE, *n*=3 individual plants. Asterisks represent significant differences between 0N and HN conditions for each genotype using Student’s *t*-test: *P<0.05, **P<0.01, ***P<0.001. (B–G) Fresh weights of the different organs measured after 35 d of N treatments. Lateral branches (LB) fresh weight per shoot fresh weight (G) were calculated. Data are presented as means ±SE, *n*=11 or 12 individual plants. Letters above the bars indicate significant differences between treatments (i.e. condition × genotype) with a multiple comparison test after Kruskal-Wallis (*P*<0.05). (H) Germination-stimulant activities of root exudates diluted 1:2. Exudates were collected at four different time points (days 9, 16, 23, and 35; Fig. 1) from group 1 and group 2 plants. Activities are presented as a percentage relative to the positive control GR24. Data are presented as means ±SE, *n*=15 (exudates from five individual plants repeated three times). (I) The same root exudate samples were diluted 1:10 and re-plated. Letters indicate significant differences between treatments (i.e. condition × genotype) using a Tukey’s test (*P*<0.05).

CS/1103P and CS/RGM were cultivated in hydroponic culture and subjected to the N treatments described in Fig. 1. For both scion/rootstock combinations, the differences in the abundance of *NIR* transcripts in grafted plants (Fig. 5A) were similar to those of cuttings (Fig. 4A). In the 0N solution, transcripts of *CCD7*, *CCD8*, and putative *MAX1* accumulated to high levels in both combinations, with greater induction in CS/RGM during week 1 (Fig. 5A). In the 0N treatment in week 2, there was no difference in the expression of *CCD7* between the two combinations, but *CCD8* and putative *MAX1* transcripts were more highly expressed in CS/RGM (Fig. 5A). In both combinations, the abundance of putative *PDR1* transcripts increased from week 1 to week 2 in 0N solution, while its expression was totally repressed under HN conditions in week 2 (Fig. 5A). The expression of the putative *PDR1* transcript showed a higher induction in the roots of CS/RGM than those of CS/1103P. These results suggest a more pronounced transcriptional response of SL-related genes to N starvation in CS/RGM than in CS/1103P.

For both combinations, the 0N solution generally inhibited plant growth (Fig. 5B–D). The root development of CS/RGM was less affected than CS/1103P during N starvation (Fig. 5B). In both HN and 0N conditions, CS/1103P tended to develop a higher LB I number/node of the main stem and a higher LB I FW/shoot FW than CS/RGM (Fig. 5F, G).

Root exudates were harvested at four time points (days 9, 16, 23 and 35; Fig. 1). Both scion/rootstock combinations produced compounds that induced the germination of *P. ramosa* L. Pomel seeds in the 0N condition, but exudates produced in the HN condition stimulated little or no seed germination (Fig. 5H, I). After 1 week of N starvation, exudates from CS/RGM induced a higher percentage of germination than exudates from CS/1103P roots (Fig. 5H, I), suggesting that the concentration of SL-like compounds in exudates from CS/RGM was higher than that from CS/1103P. The induction of germination seems to be specifically due to SLs, since exudates were also able to stimulate the germination of *Striga* seeds (Supplementary Fig. S1A). The absence of potential germination inhibitors was confirmed by the percentage of germination obtained after the addition of GR24 to the bioassays (Supplementary Fig. S1B). These bioassay experiments, together with our previous results (Fig. 4H, I), indicate that both rootstocks produced SL-like compounds in response to N starvation. Interestingly, the higher exudation of SL-like compounds from CS/RGM correlates with its stronger inhibition of shoot branching during N starvation.

### Branching patterns of woody cuttings grown in a greenhouse: effects of genotype and N supply

In order to confirm the differences in plant growth and shoot architecture observed in hydroponic culture, woody cuttings of the two rootstocks were cultivated in a greenhouse and irrigated for 45 d with an HN solution (containing 5 mM nitrate). Cuttings of both genotypes showed a significant increase in root and shoot DW over time (data not shown) with a similar relative growth rate: 0.012 g g^–1^ d^–1^ for RGM and 0.015 g g^–1^ d^–1^ for 1103P. Furthermore, total LB length/total main stem length was higher for 1103P, with an average value of 3.4 cm cm^–1^, while for RGM the main stem growth was promoted relative to the development of LB, giving a total LB length/total main stem length of only 0.89 cm cm^–1^ (Table 1). This different branching pattern could be explained by two major developmental features. First, there were more numerous LB II per LB I node, with ~4.5-fold more LB II for 1103P than for RGM for the same node number. Secondly, since the outgrowth rate of LB I per node was similar in the two genotypes, the higher number of LB in 1103P was mainly due to a higher number of nodes than in RGM (Table 1), while at the beginning of the experiment the node numbers were identical (data not shown). Furthermore, for the same plant DW or shoot DW, 1103P had more than 3-fold more LB than RGM both at the beginning and at the end of the experiment. Finally, since RGM grew longer internodes, there was no significant difference in the main stem length between the two rootstocks (Table 1).

**Table 1.** Comparison between conditions and genotypes for the growth and architectural variables of rootstock cuttings cultivated in greenhouse during 45 days under LN or HN irrigation

Growth parameters	1103P				RGM				1103P vs RGM in HN condition
	LN	HN	HN/LN	Statistical significance	LN	HN	HN/LN	Statistical significance	Statistical significance
N tester value	198.6 ± 19.3	286.4 ± 5.2	1.44	*	247.3 ± 2.6	313 ± 21.1	1.27	*	nd
Root DW (g)	3.61 ± 0.73	4.31 ± 1.09	1.19	ns	13.72 ± 1.77	11.68 ± 1.60	0.85	ns	*
Trunk DW (g)	10.81 ± 1.72	9.47 ± 1.41	0.88	ns	9.14 ± 0.73	13.12 ± 1.74	1.44	ns	ns
Shoot DW (g)	21.19 ± 2.54	29.52 ± 3.06	1.39	ns	37.69 ± 2.39	48.80 ± 3.55	1.29	*	*
Plant DW (g)	35.61 ± 4.23	42.46 ± 5.13	1.19	ns	61.53 ± 3.56	74.75 ± 4.99	1.21	ns	**
Leaf number	125.3 ± 7.6	147.2 ± 21.8	1.17	ns	57.0 ± 6.2	74.4 ± 6.8	1.31	ns	ns
Leaf surface (cm^2^)	3581 ± 398	5078 ± 224	1.42	**	4067 ± 367	5636 ± 545	1.39	*	ns
**Shoot branching parameters**									
Total main stem length (cm)	139.2 ± 28.1	221.4 ± 33.7	1.59	ns	267.7 ± 22.0	275.5 ± 33.8	1.03	ns	ns
Main stem number	1.50 ± 0.22	1.60 ± 0.27	1.07	ns	1.25 ± 0.16	1.40 ± 0.16	1.12	ns	ns
Node number	30.50 ± 5.20	40.30 ± 6.16	1.32	ns	32.62 ± 3.75	32.80 ± 4.30	1.01	ns	ns
Internode length (cm)	4.20 ± 0.37	5.42 ± 0.36	1.29	*	8.24 ± 0.46	8.20 ± 0.40	1.00	ns	***
Node number/total main stem length (cm^–1^)	0.25 ± 0.02	0.19 ± 0.01	0.76	ns	0.12 ± 0.01	0.12 ± 0.01	1.00	ns	**
Total LB number	28.90 ± 3.63	39.40 ± 2.91	1.36	ns	11.00 ± 2.08	16.50 ± 1.89	1.50	ns	***
Total LB length (cm)	490.4 ± 66.3	600.9 ± 49.5	1.23	ns	98.45 ± 16.13	203.4 ± 18.4	2.07	***	***
LB I number/node number	0.44 ± 0.05	0.49 ± 0.04	1.12	ns	0.34 ± 0.06	0.46 ± 0.04	1.36	***	ns
LB II number/LB I node number	0.19 ± 0.03	0.18 ± 0.03	0.93	ns	0.02 ± 0.01	0.04 ± 0.02	1.81	ns	***
Total LB length/total main stem length (cm cm^–1^)	4.88 ± 0.98	3.40 ± 0.56	0.70	ns	0.41 ± 0.09	0.89 ± 0.18	2.19	*	**

Data are represented as means ±SE, *n*=10 individual plants. For each variable, asterisks indicate significant differences between HN and LN conditions for each genotype as determined by a Wilcoxon test: *P<0.05, **P<0.01, ***P<0.001; ns, not significant. For comparison between both genotypes in the HN condition (last column), asterisks indicate significant differences between both genotypes as determined with Student’s *t*-test (with Bonferroni correction): *P<0.05, **P<0.01, ***P<0.001; ns, not significant; nd, not determined. For main stem number a Fisher test was used and for LB I number/node number and LB II number/LB I node number a chi-squared test was used. DW, dry weight; LB, lateral branches.

In agreement with the conclusions drawn from the hydroponic culture, these results clearly indicated that both rootstocks developed different shoot architectures, with the promotion of LB growth for 1103P, to the detriment of its main stem development, resulting in a bushier shoot architecture than RGM (see Supplementary Figs S2 and S3).

In response to LN (0.8 mM nitrate, Table 1), both genotypes presented a smaller total leaf area compared with the plants grown under HN conditions. However, a significant decrease of shoot DW was observed only for RGM. Under LN supply, neither the stem length of RGM nor its node number was impacted, whereas the growth of its LB was significantly decreased, resulting in a 2-fold reduction of the LB length per cm of main stem (Table 1). The effect of the LN treatment on 1103P resulted in plants with shorter main stems and smaller internodes than cuttings of 1103P grown in HN (Table 1). No difference could be observed between HN and LN conditions in the emergence rate of LB I per node for 1103P, resulting in an opposite branching pattern when 1103P and RGM were compared. In order to determine the plasticity of the response to HN and LN, an HN/LN ratio was calculated for each parameter in Table 1. The HN/LN ratio for total LB length/total main stem length was ~2.2 for RGM and 0.7 for 1103P.

Taken together, these results showed that the regulation of the growth of the shoot, and particularly the branching pattern, in response to N supply was different in the two genotypes. The shoot development of RGM seemed to be controlled through the regulation of LB growth, while for 1103P we observed that in the LN condition the stem development was affected (Table 1; Supplementary Fig. S3A).

### Scion branching in woody grafted plants grown in HN conditions is rootstock dependent

CS/RGM and CS/1103P were cultivated in the greenhouse for 45 d and irrigated with the same HN solution used for cuttings (5 mM nitrate). CS/1103P plants showed a higher relative growth rate (0.017 g g^–1^ d^–1^) than CS/RGM plants (0.010 g g^–1^ d^–1^). At the end of the experiment, growth parameters were not significantly different between the rootstock genotypes, except for trunk DW (Table 2). Regarding shoot branching parameters, CS/1103P plants were more branched, with a total number of LB and main stems higher than CS/RGM (Table 2; Supplementary Fig. S3B). This result is correlated with the differences in LB I number/node number.

**Table 2.** List of growth and architectural variables of CS/1103P and CS/RGM plants cultivated in greenhouse during 45 days in the HN condition

Growth parameters	CS/1103P	CS/RGM	Statistical significance
Root DW (g)	10.61 ± 1.39	11.33 ± 1.03	ns
Trunk DW (g)	14.30 ± 1.20	9.39 ± 0.52	*
Shoot DW (g)	28.87 ± 1.75	27.37 ± 2.05	ns
Plant DW (g)	57.45 ± 3.81	52.27 ± 3.04	ns
Leaf number	99.87 ± 4.82	82.33 ± 3.62	ns
Leaf surface (cm^2^)	3685 ± 177	3337 ± 155	ns
**Shoot branching parameters**			
Total main stem length (cm)	215.1 ± 12.0	177.8 ± 14.7	ns
Main stem number	2.14 ± 0.21	1.40 ± 0.13	*
Node number	52.93 ± 3.39	39.87 ± 3.22	ns
Internode length (cm)	4.04 ± 0.13	4.37 ± 0.15	ns
Node number/total main stem length (cm^–1^)	0.25 ± 0.01	0.23 ± 0.01	ns
LB I number	13.00 ± 1.01	7.00 ± 1.00	**
LB I length (cm)	56.61 ± 11.81	50.67 ± 11.44	ns
LB I number/node number	0.26 ± 0.02	0.19 ± 0.03	**
LB I length/total main stem length (cm cm^–1^)	0.28 ± 0.07	0.35 ± 0.09	ns

Data are represented as means ±SE, *n*=15 individual plants. For each variable, asterisks indicate significant differences between both genotypes as determined with Student’s *t*-test (with Bonferroni correction): *P<0.05, **P<0.01; ns, not significant. For main stem number a Fisher test was used and for the LB I number/node number a chi-squared test was used. DW, dry weight; LB, lateral branches.

These data suggest that the scion architecture in each scion/rootstock combination, grown in non-limiting nutrient conditions, depends on the rootstock genotype, whereas the growth parameters are not different. Subsequent phenotypic characterization of 1-year-old grafted plants (done the following summer) confirmed these results (Supplementary Table S2).

## Discussion

Even though grapevine rootstocks have long been known to influence scion vegetative growth, the mechanisms involved in the control of scion development are not well defined. In this study, we investigated the putative role of SLs in the rootstock-dependent control of scion development in response to N supply and highlighted the importance of the intrinsic properties of the rootstock genotypes.

### Shoot branching in response to N availability is more profoundly affected in RGM than in 1103P

Shoot branching was different between RGM and 1103P in all conditions tested, with 1103P developing bushier shoots with many branches. Since nutrient sensing plays a crucial role in regulating shoot branching, the response to changes in N supply was analysed. In hydroponic culture under N starvation conditions, shoot branching of young cuttings of both genotypes was reduced, particularly in RGM (Fig. 4). These differences were confirmed in greenhouse experiments using woody cuttings (Table 1). When the N supply was low, RGM showed reduced LB production, while the number of branches produced per node on the main stem remained the same for 1103P.

Altogether, these results showed differences in shoot branching parameters between the two genotypes and highlighted a differential response to N supply. These different patterns of shoot branching could result from differences in the perception/sensing of N availability in the roots and subsequent hormonal signalling to the shoot (Domagalska and Leyser, 2011). In addition, based on the results of the experiments with woody material, as grapevine is a perennial plant, the nutrient storage capacities of these two genotypes could also play a decisive role in their development (Richards, 1983; Zapata *et al.*, 2004; Keller, 2015).

### Possible role of SLs in shoot branching plasticity of grafted grapevine in response to N availability

Our root transcriptomic study on CS/RGM and CS/1103P grapevines grown under a heterogeneous N supply identified a gene cluster responding in a genotype-dependent manner and containing transcripts putatively involved in the biosynthesis of SLs (Cochetel *et al.*, 2017). SL biosynthesis is known to be regulated by nutrient availability and several other environmental cues (Pandey *et al.*, 2016). Overexpression of putative *CCD7* and *CCD8* from grapevine in Arabidopsis mutants of the corresponding genes (*max3-11* and *max4-1*) and overexpression of putative *CCD7*, *CCD8*, and *D27* in grapevine cells suggested that SL biosynthesis genes are conserved in grapevine (Fig. 3). Like in Arabidopsis (Turnbull *et al.*, 2002) and pea (Johnson *et al.*, 2006; Beveridge *et al.*, 2009), the expression pattern of *D27*, *CCD7*, *CCD8*, and putative *MAX1* suggests that SL biosynthesis occurs mainly in the roots of grapevine (Fig. 3). Interestingly, the expression of most SL biosynthesis genes was higher in roots of RGM than in 1103P when grafted plants were cultivated in LN conditions. For both genotypes, N starvation in hydroponic culture induced the production of exudates able to trigger the SL-inducible seed germination in the parasitic plant *P. ramosa* (Fig. 4). Expression of the genes encoding enzymes of the SL biosynthetic pathway was found to be highly induced in N starvation; this was also the case for the putative homologue of *PDR1*, which has been suggested to play a major role in SL exudation (Kretzschmar *et al.*, 2012; Sasse *et al.*, 2015) (Figs 4 and 5).

The greater exudation of SL-like compounds by CS/RGM in N starvation conditions was associated with a reduced degree of shoot branching compared with CS/1103P. The greater exudation of SL-like compounds was confirmed by other bioassays performed with *Striga* seeds. In addition, parasitic plant seed germination was not inhibited when GR24 was added to the exudates, suggesting that 1103P does not exude inhibitory compounds (Supplementary Fig. S1). These results showed that both rootstocks are able to produce putative SLs, but the specific SL compounds and their endogenous levels remain to be identified. Taken together, these results are in agreement with a putative difference of SL signalling/production between the two rootstock genotypes.

Furthermore, in plants cultivated in a greenhouse, we observed that RGM cuttings produced longer internodes than 1103P. SLs are also known to stimulate internode elongation in several species (de Saint Germain *et al.*, 2013; Lauressergues *et al.*, 2015), and this observation therefore adds further support to our hypothesis.

### Differential regulation of CS scion branching by 1103P or RGM rootstocks in non-limiting N conditions in the greenhouse

When cultivated as cuttings either in the greenhouse or in a hydroponic system, the shoot architecture differed between the two genotypes when they were grown in non-limiting N conditions (Fig. 4; Table 1). Genotype 1103P developed a bushy branching pattern, with a higher LB number and length than RGM, which showed preferential growth of the main stem. For grafted plants grown in HN conditions in the greenhouse (Table 2), we observed that 1103P promoted shoot branching by increasing the number of both LB and main stems of the CS scion, compared with RGM. This difference could contribute to the higher pruning wood weight of grapevines grafted on to 1103P in the vineyard (Zhang *et al.*, 2016). This rootstock-specific difference in shoot branching in non-limiting N conditions highlights the complexity of the signalling mechanisms established between the scion and the rootstock to control whole-plant development. The different branching patterns of the same scion grafted on to two different rootstock genotypes in non-limiting conditions revealed that molecules other than SLs, produced by the rootstock, can interact with the branching regulators produced by the scion to promote or repress shoot branching. The regulation of the balance between promoting and inhibiting signals is central in the control of scion growth by the rootstock. Our results suggest that this interaction is specific to each grafting combination and that root-derived signals play a crucial role in this process.

### Conclusion

In grafted plants, control of growth is the result of a complex signalling system established between two different genotypes (i.e. the rootstock and scion). This study showed that two rootstocks differentially regulate scion architecture, resulting in two different branching behaviours. Our results show that both genotypes produce SL-like compounds, and this correlates with the reduction of shoot branching in N-limiting conditions. Moreover, the exudation of SL-like compounds appears to be higher in the rootstock genotype that has been described to reduce scion growth, which is in agreement with a putative role of these compounds in the differential response of the two rootstocks to nutrient supply.

## Supplementary data

Supplementary data are available at *JXB* online.

Fig. S1. Germination-stimulant activities of root exudates on *Striga* seeds.

Fig. S2. Images of representative plants of each genotype after 45 days of culture in a greenhouse irrigated with HN solution (5 mM).

Fig. S3. 1103P rootstock developed more branches than RGM and this shoot branching difference is conferred to the CS scion.

Table S1. List of the primers used for RT–qPCR experiments.

Table S2. List of growth and architectural variables of 1-year-old CS/1103P and CS/RGM plants cultivated in a greenhouse.

Supplementary Figures and Tables
