# Supplementary material for: Potential contribution of strigolactones in regulating scion growth and branching in grafted grapevine in response to nitrogen availability
Source: J Exp Bot. 2018 May 30;69(16):4099–112. doi: 10.1093/jxb/ery206 (PMC6054193; doi:10.1093/jxb/ery206)
Supplement: Supplementary Figures and Tables [file ery206_suppl_supplementary_figures_tables.pdf]

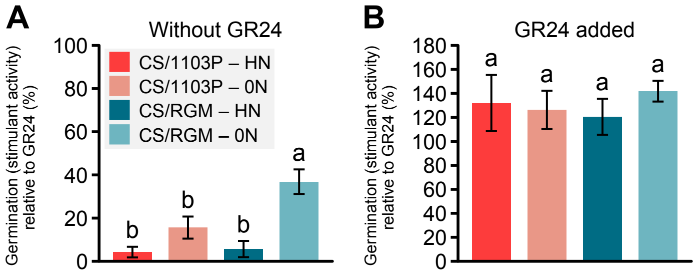

Figure S1. Germination stimulant activities of root exudates on *Striga* seeds.
Exudates of CS/1103P and CS/RGM cultivated in hydroponics were collected during the second week of culture from group 1 and group 2 plants (day 16, see Figures 1 and 4), purified on a solid phase extraction column (Strata™-X, Phenomex) and conserved in acetonitrile (water/acetonitrile 50/50, v/v, -20°C). They were diluted at 1:1000 to test germination stimulant activities on *Striga* seeds (**A**). GR24 (0.1 µM final) was added to the samples to test the presence of germination inhibitors in the exudates (**B**). Activities results are presented in percentage relative to the positive control GR24. Data are means ± SE, n=3 (exudates from 5 individual plants repeated three times). Letters indicate significant differences between treatments (*i.e.* condition x genotype) using a Tukey's test ($P < 0.05$).
The *Striga* seeds used in this experiment were collected in Soudan (2007), obtained through a collaboration with Lukáš Spíchal (Palacký University & Institute of Experimental Botany AS CR; The Czech Republic). Their germination is known to be not inducible by isothiocyanates (Lechat, 2014).

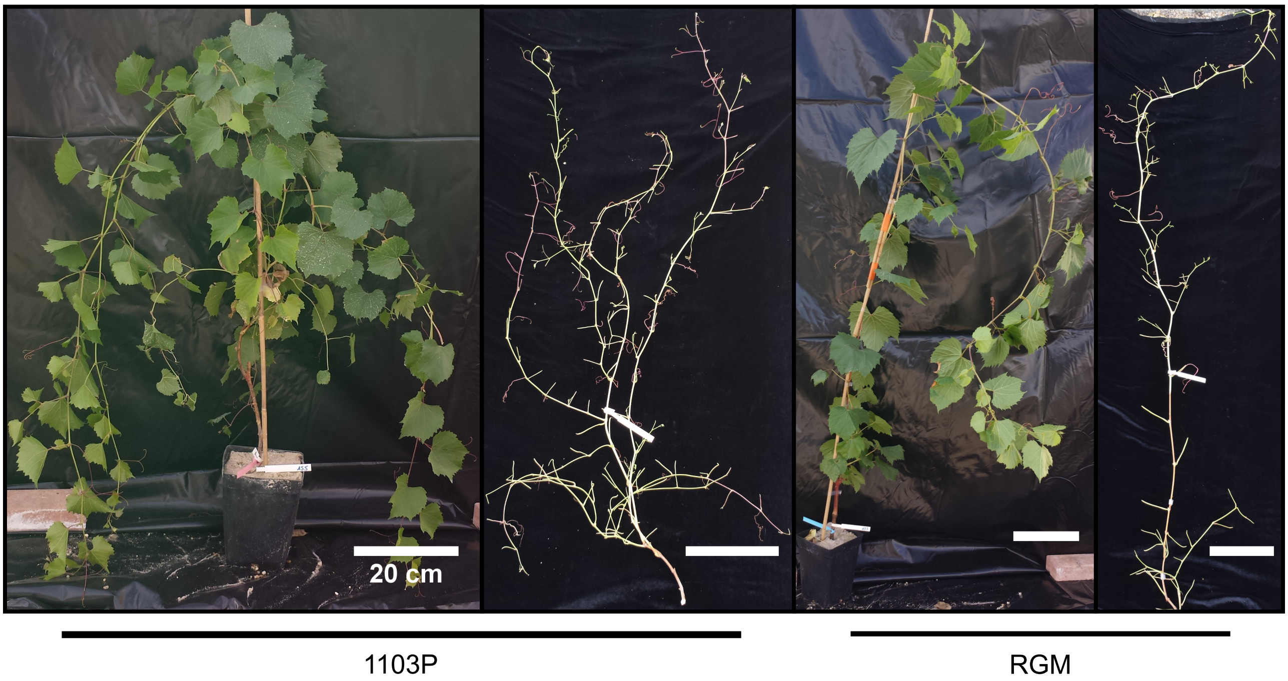

Figure S2. Images of representative plants of each genotype after 45 days of culture in greenhouse irrigated with HN solution (5 mM). For both genotypes, whole plants have been photographed (left) or only shoots without leaves (right).

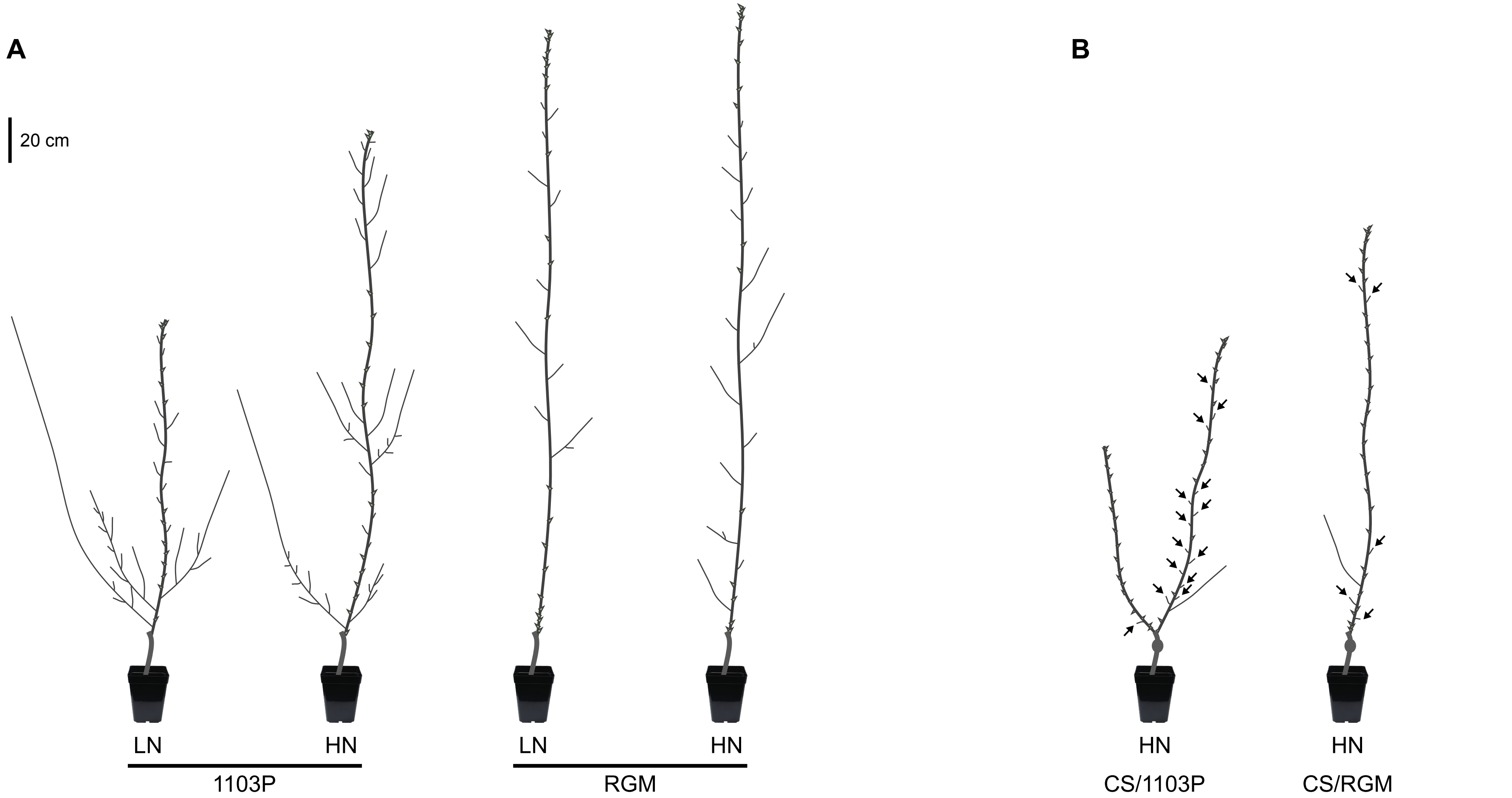

Figure S3. 1103P rootstock developed more branches than RGM and this shoot branching difference is conferred to CS scion.
A, Schematic representation of shoot architecture (without leaves) of 1103P and RGM cuttings cultivated in greenhouse and evaluated after 45 days under irrigation with LN or HN nutrient solution. **B**, Representative CS shoot architecture was also presented when grafted upon 1103P or RGM and cultivated under HN supply. The stems which grew directly from the scion wood were called « main stems ». The nodes and length of each internode along these main stems were represented. Lateral branches less than 4 cm in length were indicated using an arrow in grafted plants.

Table S1. List of the primers used for RT-qPCR experiments.

Accession number	Gene name	Function	Forward primer (5'-3')	Reverse primer (5'-3')	Amplicon length (bp)	Efficiency (%)
VIT_03s0063g00370	*NIR*	Nitrite reductase	CTTGCCGAGGAAGTGGAATGTG	GCATGTACGCCAGATCATTGATGT	80	88
VIT_11s0016g05330	*SPX1*	SPX domain-containing protein 1	ATTGCGGGCTCTGAAGGAAA	TTGAAGAGGTGGCAAGGAGA	70	88
VIT_00s0179g00330	*D27*	Dwarf 27	TGGTCAAGATCCTCCAGGGG	CAGATGGAGCAATTCACAGAGTG	96	95
VIT_15s0021g02190	*CCD7*	Carotenoid cleavage dioxygenase 7	GGAGAGATTGATGCACTTGTAGC	CCAAAAGCCATGAAATCCAA	78	91
VIT_04s0008g03380	*CCD8*	Carotenoid cleavage dioxygenase 8	CTGTGCCCTCGGAACCATTA	CCCCCTTGCGATCTCTTCAA	146	99
VIT_04s0008g01100	*MAX1*	More axillary branches 1	GAGCCATCAAACGACATCCATGA	CCTGAACCCTTAATCCAACGACT	142	95
VIT_12s0028g02140	*MAX2*	More axillary branches 2	ATTGCACCCAAAAAGACAGG	GGTGTGACAATGTGCTCTCG	78	95
VIT_18s0001g09140	*D14*	Dwarf 14	GTCAATGCCACGTGTATCTGC	TTTGCCAGGTGTCAACATCGC	97	82
VIT_17s0000g04180	*BRC1*	Branched 1	GCAAGCAGTGGAGCCCATC	AGCCTGGAAATTGAGGCTCTTG	75	90
VIT_04s0008g04790	*PDR1*	Pleiotropic drug resistance 1	AAGGGCATTCAACTTCCAAAAAAGA	TCTAGTAGCTGTAGATTCAAAAGGA	107	85
VIT_17s0000g10430	*GAPDH*	Glyceraldehyde-3-phosphate dehydrogenase	CCACAGACTTCATCGGTGACA	TTCTCGTTGAGGGCTATTCCA	70	82
VIT_12s0035g01130	*EF1γ*	Elongation factor 1 gamma	CAAGAGAAACCATCCCTAGCTG	TCAATCTGTCTAGGAAAGGAAG	91	97
Cloning						
VIT_00s0179g00330	*D27*	Dwarf 27	CTCTGTATCAGATACATGGATG	CATGTCTAGACCCTCCTTCC	794	NA
VIT_15s0021g02190	*CCD7*	Carotenoid cleavage dioxygenase 7	CCAAGGTCCAACTATGCAGC	ATGTACAAATTAACCTACTCTTTGGG	1857	NA
VIT_04s0008g03380	*CCD8*	Carotenoid cleavage dioxygenase 8	ATATCGTACTCATCTTTAATGGC	CCAGAAGTCTCTACAAATGCTTC	1731	NA
Arabidopsis transformation						
AT2G44990	*AtMAX3*	More axillary branches 3	TATGCGGTTTCGGTGGAGAG	GACACGACCGCATCGGATT	77	88
AT4G32810	*AtMAX4*	More axillary branches 4	GGTTGCTGGATCCCCAAAGA	ACAAAACCTGCACAGAGGGTA	103	85
VIT_15s0021g02190	*CCD7*	Carotenoid cleavage dioxygenase 7	CGACCTTGCCACATTTTCCG	GGGCTCACCTATGAACCTCC	104	95
VIT_04s0008g03380	*CCD8*	Carotenoid cleavage dioxygenase 8	CTGTGCCCTCGGAACCATTA	TTCCCCCTTGCGATCTCTTC	148	66
AT5G25760	*AtUBC21*	Ubiquitin-conjugating enzyme 21	TAGCATTGATGGCTCATCCT	GGCGAGGCGTGTATACATTT	119	ND
AT3G18780	*AtACT2*	Actin 2	GCACCACCTGAAAGGAAGTACA	CGATTCCTGGACCTGCCTCATC	120	ND

NA, Not Applicable; ND, Not Determined.

Table S2. List of growth and architectural variables of one-year-old CS/1103P and CS/RGM plants cultivated in greenhouse.

Growth parameters	**CS/1103P**	**CS/RGM**	**Statistical significance**
Root DW (g)	13.78 ± 1.93	15.87 ± 3.44	*ns*
Trunk DW (g)	16.48 ± 1.41	14.36 ± 1.53	*ns*
Shoot DW (g)	30.39 ± 2.64	28.66 ± 5.03	*ns*
Plant DW (g)	89.92 ± 7.84	87.21 ± 14.47	*ns*
Branching parameters			
Total main stem length (cm)	215.70 ± 16.57	185.90 ± 20.33	*ns*
Main stem number	2.80 ± 0.36	2.70 ± 0.15	*ns*
Node number	33.40 ± 2.92	36.00 ± 2.35	*ns*
Internode length (cm)	6.42 ± 0.42	4.89 ± 0.29	*ns*
Node number/Total main stem length (cm^{-1})	0.16 ± 0.01	0.21 ± 0.01	*ns*
LB I number	9.30 ± 0.62	5.00 ± 1.39	*ns*
LB I length (cm)	13.37 ± 1.90	4.67 ± 1.42	*
LB I number/Node number	0.30 ± 0.04	0.13 ± 0.04	***
LB I length/Total main stem length ($cm.cm^{-1}$)	0.07 ± 0.01	0.02 ± 0.01	*

At the end of the first growing season, a set of plants was pruned to two buds and left to over-winter in greenhouse. In the following spring, the plants were irrigated with tap water until May and then irrigated with HN solution until being harvested 2 months after bud break, corresponding to one-year-old CS/1103P and CS/RGM plants. Data are represented as means ± SE, n = 10 individual plants. For each variable, asterisks indicate significant differences between both genotypes as determined with Student test (with Bonferroni correction) (*$P < 0.05$, ***$P < 0.001$), ns: not significant. For the variable *Main stem number* a Fisher test was used and for the *LB I number/Node number* a Chi-square test was used. DW: Dry Weight; LB: Lateral Branches.
